# Supplementary material for: Yogurt Produced by Novel Natural Starter Cultures Improves Gut Epithelial Barrier In Vitro
Source: Microorganisms. 2020 Oct 15;8(10):1586. doi: 10.3390/microorganisms8101586 (PMC7602395; doi:10.3390/microorganisms8101586)
Supplement: Supplementary file 1 [file microorganisms-08-01586-s001.pdf]

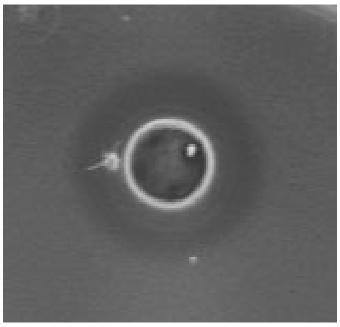

Figure S1. Inhibitory zone result of the non-proteinaceous compound activity against *Listeria monocytogenes* ATCC19111. To confirm the production of antimicrobial compounds of proteinaceous nature, crystals of pronase E were placed close to the edge of the wells.
